# The motion of point vortices on closed surfaces

**DOI:** 10.1098/rspa.2014.0890

**Published:** 2015-04-08

**Authors:** D. G. Dritschel, S. Boatto

**Affiliations:** 1 Mathematical Institute, University of St Andrews, St Andrews KY16 9SS, UK; 2 Departemento de Matemática Aplicada, Instituto de Matemática, Universidade Federal de Rio de Janeiro, Rio de Janeiro, RJ CEP 21941-909, Brazil

**Keywords:** vortex dynamics, point vortices, closed surfaces

## Abstract

We develop a mathematical framework for the dynamics of a set of point vortices on a class of differentiable surfaces conformal to the unit sphere. When the sum of the vortex circulations is non-zero, a compensating uniform vorticity field is required to satisfy the Gauss condition (that the integral of the Laplace–Beltrami operator must vanish). On variable Gaussian curvature surfaces, this results in self-induced vortex motion, a feature entirely absent on the plane, the sphere or the hyperboloid. We derive explicit equations of motion for vortices on surfaces of revolution and compute their solutions for a variety of surfaces. We also apply these equations to study the linear stability of a ring of vortices on any surface of revolution. On an ellipsoid of revolution, as few as two vortices can be unstable on oblate surfaces or sufficiently prolate ones. This extends known results for the plane, where seven vortices are marginally unstable (Thomson 1883 *A treatise on the motion of vortex rings*, pp. 94–108; Dritschel 1985 *J. Fluid Mech.* **157**, 95–134 (doi:10.1017/S0022112088003088)), and the sphere, where four vortices may be unstable if sufficiently close to the equator (Polvani & Dritschel 1993 *J. Fluid Mech.* **255**, 35–64 (doi:10.1017/S0022112093002381)).

## 1. Introduction

The original development of the theory of vortex motion goes back to Helmholtz [1] and Kelvin [2], who formulated various theorems concerning vorticity, in particular the conservation of circulation in a ‘perfect’ fluid (e.g. inviscid, incompressible and subject to forces derived from a single-valued potential) [3,4]. Kirchhoff [5] first derived the equations governed by point vortices in the two-dimensional (2D) plane. Such vortices have finite, constant circulation but singular vorticity restricted to a point. Each vortex induces a singular velocity field, entirely in the azimuthal component (with no radial component owing to zero horizontal divergence), and as a result there is no self-induced motion. Instead, vortices move in the flow field generated by all others.

The resulting model—a nonlinear Hamiltonian dynamical system—has since been studied extensively and been shown to exhibit surprisingly rich dynamics, from steady motions to chaotic motions, strongly depending on the number of vortices (for a review, see [6–8]). The model also exhibits special statistical properties for large numbers of mixed-sign vortices, noted by Onsager [9], favouring the clustering of like-signed vortices at high energies.

Interest in 2D flow on a sphere has primarily stemmed from pioneering studies of weather forecasting around 1950 [10]. The so-called ‘barotropic model’ employed was founded on the conservative transport of the scalar 2D vorticity (the vorticity component normal to the surface) by a 2D non-divergent (incompressible) ‘geostrophic’ flow. This model has remained popular ever since in idealized studies of atmospheric dynamics, though it is too simplistic to be used predictively. In fact, the equations for 2D flow on the surface of a sphere go back much earlier, to Zermelo [11]. A Lagrangian formulation of the fluid particle motion in Cartesian coordinates ***x*** with origin at the centre of the sphere was later given by Dritschel [12]. These coordinates make use of the natural isotropy of the surface, and the constraint, |***x***|= constant, is automatically satisfied by the equations of motion (this also avoids apparent polar singularities in traditional latitude–longitude coordinates [13]). These equations were then applied to study point vortex dynamics on a (non-rotating) sphere by Polvani & Dritschel [14], who also extended Thomson's [15] stability analysis of a ring of *n* equal vortices [16] finding that as few as four vortices may be unstable if located within 35.264… degrees of the equator.^1^ Boatto & Cabral [17] further extended these analyses to prove nonlinear stability, while Boatto & Simó [18,19] included the stabilizing/destabilizing effect of additional polar vortices. In particular, it was found that, for suitably chosen circulations of the polar vortices, a latitudinal ring of *n* vortices is stable everywhere, for any latitude and for all *n*.

The motion of point vortices on more general surfaces has recently attracted mathematical interest, as the more general setting allows one to better understand the role played by the surface geometry. Kimura [20] analysed constant-curvature surfaces (the sphere and the hyperboloid, respectively, with positive and negative curvature). Boatto [21] extended Kimura's analysis to prove that positive and negative curvatures have, respectively, a destabilizing and a stabilizing effect on the linear and nonlinear stability of a ring of identical vortices. More recently, Kim [22] appeared to have further extended the analysis to surfaces of variable curvature, specifically the ellipsoid of revolution, adapting the analysis of the sphere carried out by Boatto and Simó in a preprint [18]. A general theory for compact surfaces of variable curvature, however, appears to be lacking. Significant advances have been put forth by Boatto & Koiller [23,24], who clarified the role of the surface geometry on the form of the Green function and the non-singular residual Green function called the ‘Robin function’. In the absence of boundaries, the Robin function plays a role only on surfaces of variable curvature.

In this article, we develop a general theory for point vortex dynamics on a general compact, differentiable surface. This makes direct use of the Hamiltonian formalism, for which we state the general form of the vortex interaction energy for a system of *n* vortices. We apply the theory to surfaces of revolution and derive the equations of motion explicitly. We illustrate aspects of the vortex dynamics for several surfaces, and revisit the stability of a ring of vortices. We correct the previous analysis of Kim [22], who omitted the contribution due to variable surface curvature and used the incorrect area form (i.e. the incorrect Hamiltonian coordinates). This results in major differences in our conclusions.

The structure of the paper is as follows. In the next section, we derive the interaction energy for a system of *n* vortices on a closed surface. This is the Hamiltonian for the dynamical system. In §3, we show how to compute the Green function by conformal transformation, and how to properly account for the compensating vorticity field forced by the Gauss condition. In §4, we derive the equations of motion for surfaces of revolution; in §5, we illustrate the forms of various surface functions arising in the theory; and in §6, we analyse the linear stability and nonlinear evolution of a latitudinal ring of *n* identical vortices. A few conclusions are offered in §7.

## 2. Energy of a point vortex system on a compact surface

### (a) Incompressible flow induced by vorticity

We consider a general differentiable compact surface *S*. Such a surface can be viewed as embedded in $R^{3}$, and we adopt the standard notation of ***r***=(*x*_1_,*x*_2_,*x*_3_) for the position of a particle on *S*. Nevertheless, only two parameters ***s***=(*s*_1_,*s*_2_) are needed to specify a particle on a 2D surface, i.e. ***r***(***s***).^2^

Suppose that a vorticity distribution *ω*(***s***,*t*) is given on *S*,^3^ and that the corresponding flow ***u***(***s***,*t*) is incompressible, i.e. ∇⋅***u***=0. Given that *ω* is conserved following a fluid particle and d***r***/d*t*=***u***, we seek to determine the motion of an arbitrary fluid particle induced by *ω*(***s***,*t*). Note that this motion generally redistributes *ω*(***s***,*t*) so the problem is fundamentally nonlinear.

As noted by Kirchhoff [5], for planar 2D incompressible flows, we can express the velocity field in terms of a suitable differentiable function *ψ*, called the ‘streamfunction’, via

2.1 u=n×∇ψ,

where ***n*** is the local unit normal to the surface, viewed as embedded in $R^{3}$. Upon substituting this into the definition of the (scalar) vorticity, *ω*=***n*⋅**∇×***u***, the streamfunction *ψ* is seen to satisfy Poisson's equation,

2.2 Δψ=ω,

where Δ is the Laplace–Beltrami operator (Laplace's operator restricted to *S*). Then, given *ω*, the solution of this equation provides *ψ*, from which we obtain ***u*** from (2.1) and hence the particle motion and evolution of *ω*.

### (b) Inversion of the Laplace–Beltrami operator

Central to the analysis is the solution of (2.2) for *ψ*: the ‘inversion’ problem. To begin, note that for a compact surface *S* an immediate consequence of the divergence theorem is that the vorticity *ω* must integrate to zero over *S*, i.e.

2.3 $$\iint_{S}\omega (\text{s},t)\;d\Omega_{\text{s}}=0.$$

This is the so-called ‘Gauss condition’. In the integral, *dΩ*_***s***_ is the differential area element expressed in the ***s*** variables. Owing to the linearity of Poisson's equation (2.2), the general solution, to within a trivial constant, is

2.4 $$\psi (\text{s},t)=\iint_{S}G(\text{s},{\text{s}}_{o})\omega ({\text{s}}_{o},t)\;d\Omega_{{\text{s}}_{o}},$$

where *G*(***s***,***s***_*o*_) is the fundamental solution or Green function. This can be obtained from

2.5 $$\Delta G(\text{s},{\text{s}}_{o})=\delta (\text{s}-{\text{s}}_{o})-\frac{1}{A},$$

where *A* is the area of the surface *S* and *δ*(***s***−***s***_*o*_) denotes the Dirac distribution having the usual property

2.6 $$\iint_{D}\delta (\text{s}-{\text{s}}_{o})f(\text{s})\;d\Omega_{\text{s}}=\left\{ \begin{array}{ll} f({\text{s}}_{o}), & \text{if }{\text{s}}_{o}\in D\subseteq S,\\ 0, & \text{otherwise.} \end{array}\right.$$

The 1/*A* factor in (2.5) above guarantees that the Gauss condition (2.3) is satisfied and plays the role of a compensating field. The calculation of *G* for a given surface *S* is deferred to §3 below. Observe that the vorticity field *ω*(***s***−***s***_*o*_)=*δ*(***s***−***s***_*o*_)−1/*A* considered in (2.5) is the one of a point vortex, located at ***s***_*o*_, plus a specific constant uniform vorticity distribution of opposite sign.

### (c) Point vortices

Consider henceforth the special vorticity field *ω* associated with *n* point vortices, of circulations *Γ*_*j*_ and at positions ***s***_*j*_(*t*), for *j*=1,…,*n*. By linear superposition, we have

2.7 $$\omega (\text{s},t)=\sum_{j}\Gamma_{j}\left(\delta (\text{s}-{\text{s}}_{j}(t))-\frac{1}{A}\right),$$

where, for notational convenience, we define

$$\sum_{j}=\sum_{j=1}^{n}\;\text{and}\;\sum_{j\neq k}=\sum_{j=1,j\neq k}^{n}.$$

Then from (2.4), the streamfunction *ψ*(***s***,*t*) at any point ***s***≠***s***_*k*_, ∀*k* is given by

2.8 $$\psi (\text{s},t)=\sum_{k}\Gamma_{k}G(\text{s},{\text{s}}_{k}(t)).$$

Unfortunately, this cannot be used to derive the motion of the point vortices, since *ψ* is singular at each vortex and ***u*** is not defined. Instead, we use Hamilton's equations directly. The first step is to derive the interaction energy, the Hamiltonian *H*, for a system of *n* vortices on *S*. This energy is entirely kinetic, but, as we shall see, excludes a singular part which results in no motion.

The kinetic energy *E* is given by

2.9 $$E=\frac{1}{2}\iint_{S}|\text{u}(\text{s},t){|}^{2}\;d\Omega_{\text{s}}=\frac{1}{2}\iint_{S}|\nabla \psi (\text{s},t){|}^{2}\;d\Omega_{\text{s}}=-\frac{1}{2}\iint_{S}\psi (\text{s},t)\;\omega (\text{s},t)\;d\Omega_{\text{s}},$$

after integrating by parts. For point vortices, this reduces to

2.10 $$\begin{array}{rl} E & =-\frac{1}{2}\iint_{S}\psi (\text{s},t)\sum_{j}\Gamma_{j}\left(\delta (\text{s}-{\text{s}}_{j})-\frac{1}{A}\right)d\Omega_{\text{s}}\\ & =-\frac{1}{2}\sum_{j}\Gamma_{j}\psi ({\text{s}}_{j},t)+\frac{\Gamma }{2A}\iint_{S}\psi (\text{s},t)\;d\Omega_{\text{s}}, \end{array}$$

where $\Gamma =\sum_{j}\Gamma_{j}$ will be used henceforth to denote the sum of the vortex circulations.

In fluid mechanics, there is distinction to be made between a vortex, which generates the fluid dynamics, and a passive particle, which corresponds to an element of fluid that is passively advected by the velocity field produced by the vortices. For a passive particle, the equations of motion can be found by taking *E* to be the Hamiltonian *H*. The vortex motion, however, cannot be derived this way, since (2.8) is singular at each vortex position. In any case, the motion of a passive particle depends on the vortex motion, which we turn to next.

### (d) Point vortex dynamics and the Hamiltonian

We make the standard assumption, proposed by Kirchhoff [5], that a vortex behaves as a particle in the velocity field of the other vortices. However, special care must be taken to remove the self-interaction term that would result in singular behaviour. On the plane or the sphere, symmetry arguments alone lead to the conclusion that there can be no self-induced vortex motion, so that one merely has to exclude *j*=*k* from the sum in (2.8) when evaluating *ψ* at ***s***=***s***_*j*_. This renders the energy *E*—now the ‘excess energy’—finite, and we can take *H*=*E* for the Hamiltonian. Essentially, the circular streamlines around each vortex correspond to a flow with no radial component. For a general compact surface *S*, this motivates the following definition.

**Definition 2.1**

The streamfunction at the *j*th vortex is defined by the limiting process

2.11 $$\psi ({\text{s}}_{j},t)=\lim_{\text{s}\to {\text{s}}_{j}}\left(\psi (\text{s},t)-\frac{\Gamma_{j}}{2\pi }\log d(\text{s},{\text{s}}_{j})\right),$$

where *d*(***s***,***s***_*j*_) is the geodesic distance between ***s*** and ***s***_*j*_.

**Remark**

In the limit above, the (locally smooth) surface is equivalent to a portion of a planar surface, and on such a surface the streamfunction has circular streamlines. As a consequence there is no singular self-induced motion owing to the vorticity singularity.

Hence, using the expression for *ψ* in (2.8), it follows that

2.12 $$\psi ({\text{s}}_{j},t)=\sum_{k\neq j}\Gamma_{k}G({\text{s}}_{k},{\text{s}}_{j})+\Gamma_{j}R({\text{s}}_{j}),$$

where

2.13 $$R({\text{s}}_{j})=\lim_{\text{s}\to {\text{s}}_{j}}\left(G(\text{s},{\text{s}}_{j})-\frac{1}{2\pi }\log d(\text{s},{\text{s}}_{j})\right)$$

is known as the Robin function [25–27].^4^

We can now state the form of the finite, excess energy.

**Proposition 2.2**

*The excess energy of a system of* *n* *point vortices, equivalently the Hamiltonian* *H*, *is*

2.14 $$H=-\frac{1}{2}\sum_{k}\sum_{j\neq k}\Gamma_{j}\Gamma_{k}G({\text{s}}_{j},{\text{s}}_{k})-\frac{1}{2}\sum_{j}\Gamma_{j}^{2}R({\text{s}}_{j})+\frac{\Gamma }{2A}\sum_{j}\Gamma_{j}\iint_{S}G(\text{s},{\text{s}}_{j})\;d\Omega_{\text{s}}.$$

*The proof follows immediately from* (2.10) *using* (2.12).

**Remarks**

(a) This expression holds for any closed, differentiable, genus zero surface (i.e. any surface topologically equivalent to a sphere).
(b) The last term does not contribute to the dynamics as the integral of *ψ* over the whole surface is a constant (see remarks after proposition 3.1 in §3 below). This enables one to simplify the vortex Hamiltonian to
  2.15 $$H=-\frac{1}{2}\sum_{k}\sum_{j\neq k}\Gamma_{j}\Gamma_{k}G({\text{s}}_{j},{\text{s}}_{k})-\frac{1}{2}\sum_{j}\Gamma_{j}^{2}R({\text{s}}_{j}),$$
  as in [23,24], where the Green function part describes the interaction between pairs of distinct vortices, while the Robin function part can be viewed as the Hamiltonian describing the interaction of a single vortex with its uniform compensating vorticity spread across the surface. It is through *R* that a single vortex can still move on *S*. Explicit forms for *R* are given in §5. See also appendix B.
(c) Lin [25] derives a directly analogous expression for *H*, called the ‘Kirchhoff–Routh’ function (see (4.4) in that paper), for describing vortex motion in planar domains.

## 3. Calculation of the Green function by conformal transformation

We now consider a surface of revolution *S* (about the vertical *z* axis), for which much analytical progress can be made (a discussion of more general surfaces is deferred to §3d). The Cartesian coordinates ***x*** in $R^{3}$ of any point on *S* may be expressed as functions of two surface coordinates *θ* and *ϕ*, co-latitude and longitude, respectively (note: ***s***=(*θ*,*ϕ*)). For surfaces of revolution, it is sufficient to take

3.1 x=ρ(θ)cos⁡ϕ;y=ρ(θ)sin⁡ϕ;z=ζ(θ),

where *ρ*(*θ*) and *ζ*(*θ*) are specified functions of *θ*. Without loss of generality, we may take 0≤*ϕ*≤2*π* and 0≤*θ*≤*π* over *S*. Note, on a spherical surface ρ=sin⁡θ and ζ=cos⁡θ.

### (a) Conformal transformations for surfaces of revolution

We first make a conformal transformation from the punctured surface *S* (i.e. the surface without a point, *S*_p_)^5^ to the plane $R^{2}$. The first step is to compute the differential distance d*s* between two points on *S*,

3.2 $$ds^{2}=|d\text{x}{|}^{2}=dx^{2}+dy^{2}+dz^{2}=[{\left(\rho^{\prime }\right)}^{2}+{\left(\zeta^{\prime }\right)}^{2}]\;d\theta^{2}+\rho^{2}\;d\phi^{2},$$

where primes denote differentiation w.r.t. *θ*. We then consider the map $\Phi \;:\;S⟶R^{2}$ such that ***s***=(*θ*,*ϕ*)→(*r*(*θ*),*ϕ*), the usual planar polar coordinates. The goal is to find the function *r*(*θ*) in terms of which d*s*^2^ can be written as

3.3 $$ds^{2}=\lambda^{2}(dr^{2}+r^{2}\;d\phi^{2}),$$

where λ^2^(*θ*) is a conformal factor (distances are not preserved but angles are under a conformal transformation). By equating the above two expressions for d*s*^2^ in (3.2) and (3.3), we find equations defining both the conformal factor λ^2^ and the corresponding map *r*=*r*(*θ*):

3.4 $$\lambda^{2}=\frac{\rho^{2}}{r^{2}}=\frac{{\left(\rho^{\prime }\right)}^{2}+{\left(\zeta^{\prime }\right)}^{2}}{{\left(r^{\prime }\right)}^{2}}.$$

This implies

3.5 $$\frac{r^{\prime }}{r}=\frac{\sqrt{{\left(\rho^{\prime }\right)}^{2}+{\left(\zeta^{\prime }\right)}^{2}}}{\rho }\;\text{and}\;\lambda =\frac{\rho }{r}.$$

Starting from the natural boundary condition *r*(0)=0, in principle these equations can be solved given *ρ*(*θ*) and *ζ*(*θ*). Some examples are given in §5.

**Remarks**

(a) Note that r→∞ when *θ*→*π*. That is, the point *θ*=*π* is mapped to infinity, since the conformal transformation applies only to the open surface *θ*<*π*.
(b) For surfaces conformal to the sphere, as considered here, we can alternatively map directly from the sphere [23,24]. The planar map however is simpler.
(c) The expressions for infinitesimal distance in (3.2) and (3.3) (the ‘metric’ in the language of differential geometry) also provide the differential area *dΩ*_***s***_ (or ‘area form’). The latter is given by the product of distances in the perpendicular coordinate directions, i.e.
  $$d\Omega_{\text{s}}=(\lambda \;dr)\wedge (\lambda r\;d\phi )=\rho \sqrt{{\left(\rho^{\prime }\right)}^{2}+{\left(\zeta^{\prime }\right)}^{2}}\;d\theta \wedge d\phi .^{6}$$
  6The area form is an antisymmetric bilinear form [28]. Here ∧ denotes the usual exterior product [29]. To simplify notation, whenever there is no ambiguity we shall drop the symbol ∧ and denote d*θ*∧d*ϕ* simply by d*θ*d*ϕ*.
  This may also be written −*dμ*∧*dϕ* where *μ*(*θ*) is determined from
  3.6 $$\mu^{\prime }=-\rho \sqrt{{\left(\rho^{\prime }\right)}^{2}+{\left(\zeta^{\prime }\right)}^{2}}.$$
  The coordinates *μ* and *ϕ* are said to be the ‘natural’ coordinates—also called the Darboux coordinates—for the surface *S*. For a more formal approach, see Do Carmo [29].

### (b) The Green function for the punctured surface *S*_p_

We have just shown that every punctured surface can be conformally mapped into the plane $R^{2}$. That is, there exists a conformal transformation $\Phi \;:\;S_{p}\to R^{2}$, with conformal factor λ^2^(***s***), which maps *S*_p_ into $R^{2}$. On the plane, the vortex motion depends simply on the planar Green function,

3.7 $$G_{P}(\text{x},{\text{x}}_{o})=\frac{1}{2\pi }\log|\text{x}-{\text{x}}_{o}|,$$

where in this subsection ***x*** and ***x***_*o*_ denote 2D planar coordinates. *G*_*P*_ is the solution of Poisson's equation for a very special source term, a Dirac delta distribution,

3.8 $$\Delta_{\text{x}}G_{P}=\delta (\text{x}-{\text{x}}_{o}).$$

Here a subscript ***x*** is attached to Δ to distinguish the Laplace operator in $R^{2}$ from the Laplace–Beltrami operator on *S*. We continue to denote the latter by Δ throughout.

To relate *G*_*P*_ to the Green function *G*_*S*_p__ for the punctured surface *S*_p_, it proves convenient to write *G*_*P*_ explicitly in polar coordinates (*r*,*ϕ*),

3.9 $$G_{P}(\text{x},{\text{x}}_{o})=\frac{1}{4\pi }\log|\text{x}-{\text{x}}_{o}{|}^{2}=\frac{1}{4\pi }\log(r^{2}+r_{o}^{2}-2rr_{o}\cos\alpha ),$$

where $\text{x}=(r\cos\phi ,r\sin\phi ),\;{\text{x}}_{o}=(r_{o}\cos\phi_{o},r_{o}\sin\phi_{o})$ and for notational convenience *α*=*ϕ*−*ϕ*_*o*_. As shown in appendix C (and generally taken for granted), the conformal transformation $\Phi \;:\;S_{p}\to R^{2}$ maps (3.8), the equation satisfied by *G*_*P*_, into

3.10 $$\Delta G_{P}(\Phi (\text{s}),\Phi ({\text{s}}_{o}))=\lambda^{2}(\text{s})\delta (\Phi (\text{s})-\Phi ({\text{s}}_{o}))=\delta (\text{s}-{\text{s}}_{o}),$$

where ***s***, ***s***_*o*_∈*S*_p_, ***x***=*Φ*(***s***) and ***x***_*o*_=*Φ*(***s***_*o*_). We can therefore conclude that the Green function for the punctured surface is

3.11 $$G_{S_{p}}(\text{s},{\text{s}}_{o})=G_{P}(\Phi (\text{s}),\Phi ({\text{s}}_{o})),$$

and moreover *G*_*S*_p__ satisfies

3.12 $$\Delta G_{S_{p}}=\delta (\text{s}-{\text{s}}_{o})$$

with a bare Dirac distribution as its source.

**Remark**

This Green function does not apply to the closed surface *S*, since *G*_*S*_p__ does not enforce zero mean vorticity, as pointed out in §2, see (2.3). That is, the integral of *ΔG*_*S*_p__ over *S* does not vanish (in fact, it is equal to 1).

### (c) Extending the Green function to the whole surface *S*

We next show how to recover the Green function *G*_*S*_ for the whole closed surface *S*, once the Green function *G*_*S*_p__ for the punctured surface *S*_p_ is known.

**Proposition 3.1**

*The Green function for a compact surface* *S* *may be constructed from*

3.13 $$G_{S}(\text{s},{\text{s}}_{o})=G_{S_{p}}(\text{s},{\text{s}}_{o})-\bar{G}({\text{s}}_{o})-\bar{G}(\text{s}),$$

*where*

3.14 $$\bar{G}({\text{s}}_{o})=\frac{1}{A}\iint_{S_{p}}G_{S_{p}}(\text{s},{\text{s}}_{o})\;d\Omega_{\text{s}}\;\mathrm{and}\;\bar{G}(\text{s})=\frac{1}{A}\iint_{S_{p}}G_{S_{p}}(\text{s},{\text{s}}_{o})\;d\Omega_{{\text{s}}_{o}}.$$

Here, recall $d\Omega_{\text{s}}=\rho \sqrt{{\left(\rho^{\prime }\right)}^{2}+{\left(\zeta^{\prime }\right)}^{2}}\;d\theta \;d\phi =-d\mu \;d\phi$, while *A*=2*π*(*μ*(0)−*μ*(*π*)) is the total area of the surface.

**Proof.**

Applying the Laplace–Beltrami operator Δ to *G*_*S*_, we obtain

3.15 $$\begin{array}{rl} \Delta G_{S}(\text{s},{\text{s}}_{o}) & =\Delta (G_{S_{p}}(\text{s},{\text{s}}_{o})-\bar{G}({\text{s}}_{o})-\bar{G}(\text{s}))\\ & =\Delta G_{S_{p}}(\text{s},{\text{s}}_{o})-\frac{1}{A}\iint_{S}(\Delta G_{S_{p}}(\text{s},{\text{s}}_{o}))\;d\Omega_{{\text{s}}_{o}}\\ & =\Delta G_{S_{p}}(\text{s},{\text{s}}_{o})-\frac{1}{A}=\delta (\text{s}-{\text{s}}_{o})-\frac{1}{A}, \end{array}$$

since $\bar{G}({\text{s}}_{o})$ is independent of ***s***. Hence, integrating over *S*, we find

3.16 $$\iint_{S}\Delta G_{S}(\text{s},{\text{s}}_{o})\;d\Omega_{\text{s}}=0,$$

which is exactly the condition we require for the Green function on *S*. As verified in appendix A, the extra terms added to *G*_*S*_p__ in (3.13) cancel the singularities in *G*_*S*_p__ at *θ*=*π* and *θ*_*o*_=*π*, so that *G*_*S*_ is regular at these points. That is, *ΔG*_*S*_ is finite at *θ*=*π* and *θ*_*o*_=*π*, justifying taking the domain of integration in (3.16) as the whole compact surface *S*. Hence, *G*_*S*_ is the Green function for *S*, to within an unimportant constant. ▪

**Remarks**

Properties of the Green function.

(a) The function *G*_*S*_ is symmetric with respect to its arguments, i.e. *G*_*S*_(***s***,***s***_*o*_)=*G*_*S*_(***s***_*o*_,***s***).
(b) Two extra terms are included to maintain the required symmetry of *G*_*S*_. The second term on the r.h.s. is a function only of ***s***_*o*_, while the third is a function only of ***s***.
(c) It follows from (3.13) and (3.14) that
  3.17 $$\begin{array}{rl} \iint_{S}G_{S}(\text{s},{\text{s}}_{o})\;d\Omega_{\text{s}} & =\iint_{S}[G_{S_{p}}(\text{s},{\text{s}}_{o})-\bar{G}({\text{s}}_{o})-\bar{G}(\text{s})]\;d\Omega_{\text{s}}\\ & =\iint_{S}G_{S_{p}}(\text{s},{\text{s}}_{o})\;d\Omega_{\text{s}}-\bar{G}({\text{s}}_{o})\;A-\iint_{S}\bar{G}(\text{s})\;d\Omega_{\text{s}}\\ & =\bar{G}({\text{s}}_{o})A-\bar{G}({\text{s}}_{o})A-\iint_{S}\bar{G}(\text{s})\;d\Omega_{\text{s}}=-\iint_{S}\bar{G}(\text{s})\;d\Omega_{\text{s}} \end{array}$$
  is just a constant. In the second line, we have used (3.14) to evaluate the integral over *G*_*S*_p__. Strictly speaking, $\bar{G}$ is found by integrating over the punctured surface *S*_p_, but the additional point in *S* contributes nothing to the integral.

**Proposition 3.2**

*The function* *G*_*S*_ *defined in* (3.13) *satisfies*

3.18 $$\lim_{\theta \to \pi }G_{S}(\text{s},{\text{s}}_{o})=-\frac{1}{2\pi }\log(r_{o})\;\mathrm{and}\;\lim_{\theta_{o}\to \pi }G_{S}(\text{s},{\text{s}}_{o})=-\frac{1}{2\pi }\log(r)$$

*and therefore is valid over the whole surface* *S*, *not just on the punctured surface*, *S*_p_=*S*−{*θ*=*π*}.

A simple proof is given in appendix A.

**Remark**

For a spherical surface, discussed in §5a below, one can show that this construction gives the correct form for *G*_*S*_, proportional to the chord distance between ***s*** and ***s***_*o*_.

### (d) General surfaces

For more general surfaces (of genus zero) without rotational symmetry such as a tri-axial ellipsoid, we can in principle construct the Green function in the same way as outlined for surfaces of revolution. The procedure is as follows:

(1) find a conformal transformation between the surface *S* and the plane $R^{2}$;
(2) make use of the known Green function *G*_*S*_p__ for the punctured surface; and
(3) correct for the condition of zero total vorticity, i.e. set $G_{S}(\text{s},{\text{s}}_{o})=G_{S_{p}}(\text{s},{\text{s}}_{o})-\bar{G}(\text{s})-\bar{G}({\text{s}}_{o})$.

The recipe is clear, though the devil is in the detail!

### (e) The Hamiltonian

The Hamiltonian *H* derived in §2 simplifies when we make use of the Green function

$$G_{S}(\text{s},{\text{s}}_{o})=G_{S_{p}}(\text{s},{\text{s}}_{o})-\bar{G}(\text{s})-\bar{G}({\text{s}}_{o}),$$

derived just above. To simplify the notation, from now on we shall suppress the subscript *S* and simply use *G*(***s***,***s***_*o*_) for the Green function on the surface *S*.

As previously remarked, the last term in the Hamiltonian *H* in (2.14) is just a constant, independent of the vortex positions, so does not contribute to the vortex dynamics. (This is explicitly shown using the expression for *G* above in the remarks after proposition 3.1, in (3.17).) It is sufficient therefore to consider the simplified Hamiltonian given in (2.15).

For a surface of revolution, the Robin function *R*(***s***_*j*_) defined in (2.13) also simplifies considerably. In the expression for *R*, in the limit ***s***→***s***_*j*_, the geodesic distance *d*(*s*,*s*_*j*_) has the same form as the infinitesimal distance, d*s*, introduced at the beginning of §3a. Explicitly,

3.19 $$\begin{array}{rl} \lim_{\text{s}\to {\text{s}}_{j}}d(\text{s},{\text{s}}_{j}) & =\lambda ({\text{s}}_{j})\lim_{\text{s}\to {\text{s}}_{j}}\sqrt{{\left(r-r_{j}\right)}^{2}+r_{j}^{2}{\left(\phi -\phi_{j}\right)}^{2}} \end{array}$$

3.20 $$\begin{array}{rl} & =\lambda ({\text{s}}_{j})\lim_{\text{s}\to {\text{s}}_{j}}\sqrt{r^{2}+r_{j}^{2}-2rr_{j}\cos(\phi -\phi_{j})}, \end{array}$$

where *r*=*r*(*θ*) and *r*_*j*_=*r*(*θ*_*j*_), and the second identical expression is introduced to draw a parallel to *G*_*S*_p__:

$$G_{S_{p}}(\text{s},{\text{s}}_{j})=G_{P}(\Phi (\text{s}),\Phi ({\text{s}}_{j}))=G_{P}(\text{x},{\text{x}}_{j})=\frac{1}{4\pi }\log(r^{2}+r_{j}^{2}-2rr_{j}\cos(\phi -\phi_{j})),$$

using (3.10) and (3.11), with x=(rcos⁡ϕ,rsin⁡ϕ), ${\text{x}}_{j}=(r_{j}\cos\phi_{j},r_{j}\sin\phi_{j})$, as before. Now it is evident that the log singularities in *R* cancel, leaving

$$\begin{array}{rl} R({\text{s}}_{j}) & =\lim_{\text{s}\to {\text{s}}_{j}}\left(G_{S_{p}}(\text{s},{\text{s}}_{j})-\bar{G}(\text{s})-\bar{G}({\text{s}}_{j})-\frac{1}{2\pi }\log d(\text{s},{\text{s}}_{j})\right)\\ & =-\frac{1}{2\pi }\log[\lambda ({\text{s}}_{j})]-2\bar{G}({\text{s}}_{j}), \end{array}$$

where λ(***s***_*j*_)=*ρ*(*θ*_*j*_)/*r*(*θ*_*j*_) depends only on *θ*_*j*_ for a surface of revolution. Furthermore, as shown in (A.5), $\bar{G}({\text{s}}_{j})$ depends only on *θ*_*j*_, so that the Robin function *R* also depends only on *θ*_*j*_.

**Remark**

For a bounded 2D domain, the Robin function *R* has the exact same dependence on the conformal factor λ arising in the conformal transformation mapping the boundary to the unit circle (e.g. §3.3 in [8] and also appendix C). However, there is no analogue of the function $\bar{G}$, which arises here because the surface *S* is closed, as shown in proposition 3.1.

## 4. Equations of motion

Given the Hamiltonian *H*(***s***_1_,***s***_2_,…***s***_*n*_), the equations of motion may be generally written

4.1 $$\Gamma_{k}{\dot{s}}_{k}={\tilde{\text{J}}}_{2}\nabla_{{\text{s}}_{k}}H\;(k=1,\ldots ,n),$$

where a dot indicates a time derivative, and

4.2 $${\tilde{\text{J}}}_{2}=\left(\begin{array}{cc} 0 & 1\\ -1 & 0 \end{array}\right)$$

is the standard skew-symmetric 2×2 matrix.

### (a) Surfaces of revolution

Surfaces of revolution have a natural ‘position-momentum’ canonical pair (*q*_*k*_,*p*_*k*_)=(*ϕ*_*k*_,*Γ*_*k*_*μ*_*k*_) for each vortex *k*, where

4.3 $${\text{s}}_{k}=(\phi_{k},\mu_{k})=\left(q_{k},\frac{p_{k}}{\Gamma_{k}}\right)\;k=1,\ldots ,n.$$

Here *μ* and *ϕ* are the ‘natural’ coordinates for which the area element d*Ω*_***s***_=−d*μ* d*ϕ* (see remark (c) at the end of §3a). Moreover, incompressibility in these coordinates reduces to

4.4 $$\nabla \cdot \text{u}=\frac{\partial \dot{\mu }}{\partial \mu }+\frac{\partial \dot{\phi }}{\partial \phi }=0$$

(in planar polar coordinates, for example, *μ*=*r*^2^/2; see [30]). Hence, there exists a streamfunction *ψ* such that

4.5 $$\dot{\phi }=\frac{\partial \psi }{\partial \mu };\;\dot{\mu }=-\frac{\partial \psi }{\partial \phi }.$$

For the point vortex dynamics, the very same equations apply for each vortex *k*, and the streamfunction is just that given in (2.12), i.e. the regular part of the streamfunction evaluated at the vortex position. Equivalently, and consistently, ${\dot{\phi }}_{k}$ and ${\dot{\mu }}_{k}$ can be obtained from Hamilton's equations (4.1), identifying *ϕ*_*k*_ as the ‘position’ and *Γ*_*k*_*μ*_*k*_ as the ‘momentum’:

4.6 $${\dot{\phi }}_{k}=\frac{1}{\Gamma_{k}}\frac{\partial H}{\partial \mu_{k}};\;\Gamma_{k}{\dot{\mu }}_{k}=-\frac{\partial H}{\partial \phi_{k}}.$$

In fact, the identification of *Γ*_*k*_*μ*_*k*_ with ‘momentum’ is appropriate since it is immediate to show that

4.7 $$M=\sum_{k}\Gamma_{k}\mu_{k}$$

is conserved owing to the rotational symmetry of *H* (see below). This is just the angular impulse.

We defer the explicit calculation of the partial derivatives in (4.6) and first introduce a transformation of the equations of motion that is beneficial for their numerical implementation. We begin by rewriting the equations of motion (4.6) in the coordinates (*ϕ*,*θ*), for which

4.8 $${\dot{\phi }}_{k}=\frac{1}{\Gamma_{k}\mu^{\prime }(\theta_{k})}\frac{\partial H}{\partial \theta_{k}};\;{\dot{\theta }}_{k}=-\frac{1}{\Gamma_{k}\mu^{\prime }(\theta_{k})}\frac{\partial H}{\partial \phi_{k}},$$

where $\mu^{\prime }=-\rho \sqrt{{\left(\rho^{\prime }\right)}^{2}+{\left(\zeta^{\prime }\right)}^{2}}$; see (3.6). Note that *μ*′(*θ*)<0 everywhere except at the poles *θ*=0 and *π* where *μ*′=0. There, ${\dot{\phi }}_{k}$ is singular because arbitrarily rapid angular variations occur for vortex trajectories crossing through either pole (${\dot{\theta }}_{k}$, however, is finite).

The singularity in ${\dot{\phi }}_{k}$ can be avoided using instead three coordinates, analogous to Cartesian coordinates, constrained to the surface [12],

4.9 X=sin⁡θcos⁡ϕ,Y=sin⁡θsin⁡ϕandZ=cos⁡θ,

satisfying $X^{2}+Y^{2}+Z^{2}=1$. Their time derivatives are given by

4.10 $${\dot{X}}_{k}=-Z_{k}v_{k}\cos\phi_{k}-u_{k}\sin\phi_{k},\;{\dot{Y}}_{k}=-Z_{k}v_{k}\sin\phi_{k}+u_{k}\cos\phi_{k}\;\mathrm{and}\;{\dot{Z}}_{k}=\sin\theta_{k}v_{k},$$

where $u_{k}=\sin\theta_{k}{\dot{\phi }}_{k}$ is the ‘azimuthal’ velocity and $v_{k}=-{\dot{\theta }}_{k}$ is the ‘meridional’ velocity—both of which are finite at all points, including the poles. While these equations are redundant, they are regular everywhere and convenient for numerical implementation.

We next turn to the calculation of the partial derivatives ∂*H*/∂*ϕ*_*k*_ and ∂*H*/∂*θ*_*k*_ in (4.6). To this end, note that, for a surface of revolution,

4.11 $$G({\text{s}}_{j},{\text{s}}_{k})=\frac{1}{4\pi }\log(r_{j}^{2}+r_{k}^{2}-2r_{j}r_{k}\cos(\phi_{j}-\phi_{k}))-\bar{G}(\theta_{j})-\bar{G}(\theta_{k}),$$

where *r*_*j*_=*ρ*(*θ*_*j*_)/λ(*θ*_*j*_) and *r*_*k*_=*ρ*(*θ*_*k*_)/λ(*θ*_*k*_), while

4.12 $$R({\text{s}}_{k})=R(\theta_{k})=-\frac{1}{2\pi }\log\lambda (\theta_{k})-2\bar{G}(\theta_{k}).$$

To simplify notation, we suppress the subscript *k* and change *j* to *o*. Functions evaluated at *θ* are subscript free, while those evaluated at *θ*_*o*_ acquire a subscript *o*.

It proves useful to eliminate $\bar{G}$ using the definition of *R* above in (4.12):

$$-\bar{G}=\frac{1}{4\pi }\log\lambda +\frac{1}{2}R.$$

Then,

4.13 $$\begin{array}{rl} G & =\frac{1}{4\pi }\log(\lambda \lambda_{o}(r^{2}+r_{o}^{2}-2rr_{o}\cos\alpha ))+\frac{1}{2}(R+R_{o})\\ & =\frac{1}{4\pi }\log\left(\frac{\lambda_{o}}{\lambda }\rho^{2}+\frac{\lambda }{\lambda_{o}}\rho_{o}^{2}-2\rho \rho_{o}\cos\alpha \right)+\frac{1}{2}(R+R_{o}) \end{array}$$

after using *r*=*ρ*/λ and *r*_*o*_=*ρ*_*o*_/λ_*o*_ (here, as before, *α*=*ϕ*−*ϕ*_*o*_). We next introduce the function

4.14 $$\xi =\frac{\rho^{2}}{\lambda },$$

which is finite for all *θ*. Then, since *R* depends only on *θ*, the partial derivative of *G* w.r.t. *ϕ* is given by

4.15 $$\frac{\partial G}{\partial \phi }=\frac{1}{4\pi }\cdot \frac{2\rho \rho_{o}\sin\alpha }{\lambda_{o}\xi +\lambda \xi_{o}-2\rho \rho_{o}\cos\alpha },$$

whose numerator and denominator involve only functions of *θ* and *θ*_*o*_ that are finite over the entire range [0,*π*]. (For a sphere, see §5a below, the denominator is the distance in $R^{3}$ between two points on the surface.) The antisymmetry of this expression, i.e. ∂*G*(***s***,***s***_*o*_)/∂*ϕ*=−∂*G*(***s***_*o*_,***s***)/∂*ϕ*_*o*_, arises from the rotational symmetry of *S*. A direct consequence of this is conservation of angular impulse (4.7).

The partial derivative of *G* w.r.t. *θ* is

4.16 $$\frac{\partial G}{\partial \theta }=\frac{1}{4\pi }\cdot \frac{\lambda_{o}\xi^{\prime }+\lambda^{\prime }\xi_{o}-2\rho^{\prime }\rho_{o}\cos\alpha }{\lambda_{o}\xi +\lambda \xi_{o}-2\rho \rho_{o}\cos\alpha }+\frac{1}{2}R^{\prime }.$$

Here again the numerator and denominator involve only finite functions of *θ* and *θ*_*o*_. The derivatives *ξ*′ and λ′ are given explicitly by

4.17 $$\xi^{\prime }=\frac{\rho }{\lambda }\left(\rho^{\prime }+\sqrt{{\left(\rho^{\prime }\right)}^{2}+{\left(\zeta^{\prime }\right)}^{2}}\right)\;\mathrm{and}\;\lambda^{\prime }=\frac{\lambda }{\rho }\left(\rho^{\prime }-\sqrt{{\left(\rho^{\prime }\right)}^{2}+{\left(\zeta^{\prime }\right)}^{2}}\right)$$

and, notably, they vanish at *θ*=0 and *π* and are finite elsewhere. In ∂*G*/∂*θ*, only the derivative of the Robin function *R* is needed, and after some manipulation we find

4.18 $$R^{\prime }=-\frac{1}{2\pi \rho }\left[\rho^{\prime }-\frac{\mu }{\mu (0)}\sqrt{{\left(\rho^{\prime }\right)}^{2}+{\left(\zeta^{\prime }\right)}^{2}}\right],$$

where we have taken *μ*(0)=*A*/(4*π*) without loss of generality (then *μ*(*π*)=−*μ*(0)). Note that *R*′, like *ξ*′ and λ′, vanishes at *θ*=0 and *π*.

Putting everything together (and noting that distinct pairs of integers appear twice in the double sum involved in *H*; see (2.15)), we can now write down the explicit form of the azimuthal and meridional velocity components, restoring the *j* and *k* subscripts:

4.19 $$\begin{array}{rl} u_{k} & =\sin\theta_{k}{\dot{\phi }}_{k}\;=\;\frac{\Gamma }{2\pi }\beta_{k}+\chi_{k}\sum_{j\neq k}\frac{\Gamma_{j}}{2\pi }\cdot \frac{\lambda_{j}\xi_{k}^{\prime }+\lambda_{k}^{\prime }\xi_{j}-2\rho_{k}^{\prime }\rho_{j}\cos(\phi_{k}-\phi_{j})}{\lambda_{j}\xi_{k}+\lambda_{k}\xi_{j}-2\rho_{k}\rho_{j}\cos(\phi_{k}-\phi_{j})}\\ \text{and}\;v_{k} & =-{\dot{\theta }}_{k}=\tau_{k}\sum_{j\neq k}\frac{\Gamma_{j}}{2\pi }\cdot \frac{\rho_{j}\sin(\phi_{k}-\phi_{j})}{\lambda_{j}\xi_{k}+\lambda_{k}\xi_{j}-2\rho_{k}\rho_{j}\cos(\phi_{k}-\phi_{j})}, \end{array}\}$$

where we have introduced three additional functions

4.20 $$\tau =\frac{1}{\sqrt{{\left(\rho^{\prime }\right)}^{2}+{\left(\zeta^{\prime }\right)}^{2}}},\;\chi =\frac{\tau \sin\theta }{2\rho }$$

and

4.21 $$\beta =2\pi \chi R^{\prime }=2\pi \sin\theta \frac{\tau R^{\prime }}{2\rho }=\frac{\sin\theta }{2\rho^{2}}\left(\frac{\mu }{\mu (0)}-\frac{\rho^{\prime }}{\sqrt{{\left(\rho^{\prime }\right)}^{2}+{\left(\zeta^{\prime }\right)}^{2}}}\right),$$

which are finite for all *θ*, and moreover *β*(0)=*β*(*π*)=0.

**Remark**

A single vortex rotates about the *z* axis at the rate $\Gamma_{k}\beta_{k}/(2\pi \sin\theta_{k})$, and the sums in (4.19) are absent. Then, only the Robin function contributes to the dynamics. This motion arises due to variable surface curvature—a single vortex does not move on a sphere. In the limit *θ*→0, we find $\beta /\sin\theta \to {\left(\zeta^{\prime \prime }(0)/2{\left(\rho^{\prime }(0)\right)}^{2}\right)}^{2}-\pi /A$, whereas in the limit *θ*→*π*, we find $\beta /\sin\theta \to \pi /A-{\left(\zeta^{\prime \prime }(\pi )/2{\left(\rho^{\prime }(\pi )\right)}^{2}\right)}^{2}$. These expressions vanish for a spherical surface. Notably, when the total circulation vanishes (*Γ*=0), there is no contribution from the Robin function.

## 5. Examples

### (a) The unit sphere $S^{2}$

**Figure 1.**
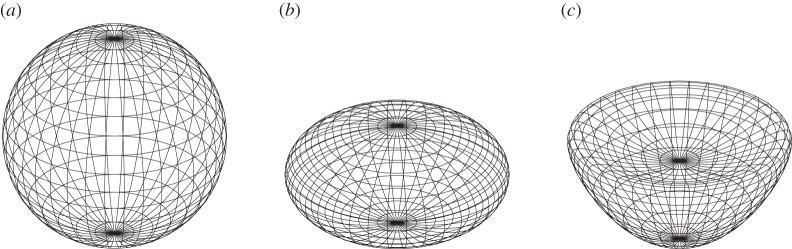
(*a*) A sphere, (*b*) an ellipsoid of revolution and (*c*) a bean-shaped surface with parameters *a*=0.6 and *b*=0.4; see (3.1) and (5.13). The three figures are from an orthographic perspective, viewed from 60° co-latitude and 15° longitude.

In (3.1), the unit sphere, see figure 1*a*, is described by the functions ρ=sin⁡θ and ζ=cos⁡θ. The planar radius *r*(*θ*) satisfies

5.1 $$\frac{r^{\prime }}{r}=\frac{\sqrt{{\left(\rho^{\prime }\right)}^{2}+{\left(\zeta^{\prime }\right)}^{2}}}{\rho }=\frac{1}{\sin\theta },$$

which can be integrated starting from *r*(0)=0 to give

5.2 $$r(\theta )=\frac{\sin\theta }{1+\cos\theta }=\sqrt{\frac{1-\cos\theta }{1+\cos\theta }},$$

in terms of which the conformal factor is given by

5.3 $$\lambda =\frac{\rho }{r}=1+\cos\theta .$$

The meridional coordinate satisfies

5.4 $$\mu^{\prime }=-\rho \sqrt{{\left(\rho^{\prime }\right)}^{2}+{\left(\zeta^{\prime }\right)}^{2}}=-\sin\theta ,$$

so that μ=cos⁡θ, which is the true axial Cartesian coordinate *z*. Note *A*=4*π*.

Next, we turn to the calculation of $\bar{G}(\theta )$. From §3a, this is given by

5.5 $$\begin{array}{rl} 4\pi \bar{G}(\theta ) & =\log r^{2}(\theta )+\int_{\theta }^{\pi }(\mu (\theta_{o})-\mu (\pi ))\frac{r^{\prime }(\theta_{o})}{r(\theta_{o})}\;d\theta_{o}\\ & =\log\left(\frac{1-\cos\theta }{1+\cos\theta }\right)+\int_{\theta }^{\pi }\frac{\cos\theta_{o}+1}{\sin\theta_{o}}\;d\theta_{o}\\ & =\log\left(\frac{1-\cos\theta }{1+\cos\theta }\right)+\int_{\theta }^{\pi }\frac{\sin\theta_{o}}{1-\cos\theta_{o}}\;d\theta_{o}\\ & =\log\left(\frac{1-\cos\theta }{1+\cos\theta }\right)+{\left[\log(1-\cos\theta_{o})\right]}_{\theta }^{\pi }\\ & =-\log\left(\frac{1+\cos\theta }{2}\right), \end{array}$$

which is singular only as *θ*→*π*, as anticipated from the discussion in §3a. The Green function, therefore, takes the form

5.6 $$\begin{array}{rl} G(\text{s},{\text{s}}_{o}) & =\frac{1}{4\pi }\log(r^{2}+r_{o}^{2}-2rr_{o}\cos(\phi -\phi_{o}))-\bar{G}(\theta )-\bar{G}(\theta_{o})\\ & =\frac{1}{4\pi }\log\left(\frac{1-\cos\theta }{1+\cos\theta }+\frac{1-\cos\theta_{o}}{1+\cos\theta_{o}}-2\sqrt{\frac{1-\cos\theta }{1+\cos\theta }\cdot \frac{1-\cos\theta_{o}}{1+\cos\theta_{o}}}\cos(\phi -\phi_{o})\right)\\ & \;+\frac{1}{4\pi }\log\left(\frac{\left(1+\cos\theta )(1+\cos\theta_{o}\right)}{4}\right)\\ & =\frac{1}{4\pi }\log(2-2\cos\theta \cos\theta_{o}-2\sqrt{\left(1-\cos^{2}\theta )(1-\cos^{2}\theta_{o}\right)}\cos(\phi -\phi_{o}))-\frac{1}{4\pi }\log4\\ & =\frac{1}{2\pi }\log\left(\frac{|\text{x}-{\text{x}}_{o}|}{2}\right), \end{array}$$

where |***x***−***x***_*o*_| is the chord distance (usual distance in $R^{3}$) between ***x*** and ***x***_*o*_. This is the classical result [12].

The Robin function given by

5.7 $$R(\theta )=-\frac{1}{2\pi }\log\lambda (\theta )-2\bar{G}(\theta )=-\frac{\log2}{2\pi }$$

is just a constant, and therefore plays no role in the dynamics.

Finally, the equations of motion can be expressed simply in terms of the Cartesian coordinates ***x***_*k*_ of each vortex (one needs only to derive the equation for ${\dot{z}}_{k}$ and use symmetry to obtain the others for ${\dot{x}}_{k}$ and ${\dot{y}}_{k}$; see [12]):

5.8 $${\dot{\text{x}}}_{k}=\frac{1}{4\pi }\sum_{j\neq k}\Gamma_{j}\frac{{\text{x}}_{j}\times {\text{x}}_{k}}{1-{\text{x}}_{j}\cdot {\text{x}}_{k}}.$$

### (b) The ellipsoid of revolution

Consider the ellipsoid of revolution (or spheroid), as in figure 1*b*, having a vertical to horizontal aspect ratio *b*. In (3.1), this surface is defined by

5.9 ρ=sin⁡θandζ=bcos⁡θ.

The forms of the various surface functions such as *μ*(*θ*) and λ(*θ*) are markedly more complex than in the case of the sphere. Defining $q=\tau^{-1}=\sqrt{{\left(\rho^{\prime }\right)}^{2}+{\left(\zeta^{\prime }\right)}^{2}}=\sqrt{\cos^{2}\theta +b^{2}\sin^{2}\theta }$, the meridional coordinate *μ* takes the form

5.10 $$\mu (\theta )=\frac{q\cos\theta }{2}+\left\{ \begin{array}{ll} \frac{b^{2}}{2\sqrt{1-b^{2}}}\ln\left(\frac{q+\sqrt{1-b^{2}}\cos\theta }{b}\right): & b<1\\ \frac{b^{2}}{2\sqrt{b^{2}-1}}\sin^{-1}\left(\frac{\sqrt{b^{2}-1}\cos\theta }{b}\right): & b>1. \end{array}\right.$$

Note that the surface area *A*=4*πμ*(0). The conformal factor λ is more complicated still, and we find

5.11 $$\begin{array}{r} \lambda (\theta )=b(1+\cos\theta ){\left(\frac{q+b^{2}+(1-b^{2})\cos\theta }{q+b^{2}-(1-b^{2})\cos\theta }\right)}^{1/2}\times \left\{ \begin{array}{ll} {\left(\frac{q+\sqrt{1-b^{2}}\cos\theta }{1+\sqrt{1-b^{2}}}\right)}^{-\sqrt{1-b^{2}}}: & b<1\\ e^{-\sqrt{b^{2}-1}\cos^{-1}(\sqrt{b^{2}-1}\cos\theta /b)}: & b>1, \end{array}\right. \end{array}$$

where we have taken λ(0)=2 without loss of generality (any value could be taken as only ratios of λ appear in the dynamical equations (4.19)). These forms of *μ* and λ, for both *b*<1 and *b*>1, have been verified by direct numerical integration to machine precision accuracy.

**Figure 2.**
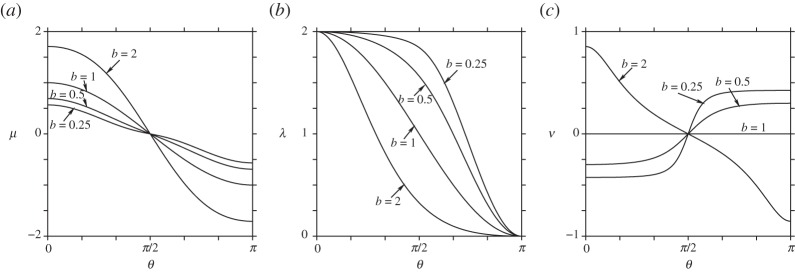
The form of various surface functions for an ellipsoid: (*a*) the meridional coordinate *μ*(*θ*), (*b*) the conformal factor λ(*θ*) and (*c*) the rotation rate *ν*(*θ*) of a single vortex with *Γ*=2*π*.

Figure 2 shows the form of *μ*(*θ*) and λ(*θ*), as well as *ν*(*θ*), the rotation rate of a single vortex of circulation *Γ*=2*π* about the axis of symmetry. The rotation rate is given in terms of *β*(*θ*) from (4.21), specifically

5.12 $$\nu =\frac{\beta }{\sin\theta }=\frac{1}{2\rho^{2}}\left[\frac{\mu }{\mu (0)}-\frac{\rho^{\prime }}{\sqrt{{\left(\rho^{\prime }\right)}^{2}+{\left(\zeta^{\prime }\right)}^{2}}}\right].$$

The meridional coordinate *μ*(*θ*) is antisymmetric about the equator *θ*=*π*/2. In general, *μ* varies more strongly with *θ* as *b* increases (as the shape becomes more prolate), which is to be expected since 2*π*(*μ*(0)−*μ*) measures the surface area from the north pole *θ*=0 to any ‘co-latitude’ *θ*. Taller shapes have greater surface area than shorter ones. The conformal factor λ decreases to zero more rapidly for prolate shapes than for oblate ones. As *b*→0, λ develops an extensive flat region where λ≈2. The rotation rate of a single vortex *ν*, like *μ*, is antisymmetric about the equator *θ*=*π*/2. Positive-signed vortices on prolate shapes *b*>1 rotate cyclonically (counter-clockwise) about the north pole if they are located in the northern hemisphere. In the southern hemisphere, they rotate the opposite way. The fastest rotation rates occur at the poles. The situation is reversed for oblate shapes *b*<1, while, for a sphere *b*=0, a single vortex is stationary.

Notably, the motion of a vortex dipole (with *Γ*=*Γ*_1_+*Γ*_2_=0) appears to be quasi-periodic in general. The average position of the dipole does not generally form closed orbits, unlike in the case of a spherical surface on which all orbits are great circles.

### (c) The bean surface

In (3.1), consider the surface defined by

5.13 $$\rho =\sin\theta \;\mathrm{and}\;\zeta =a\sin^{2}\theta +b\cos\theta .$$

When *a*=0 and *b*=1, we recover the sphere, while for general *b* the surface is a spheroid (an ellipsoid with semi-axis lengths 1, 1 and *b*). When *a*>0, the upper part of the surface is dented inwards, producing a region of negative curvature—a ‘bean’ shape. An example is provided in figure 1*c* for the parameters *a*=0.6 and *b*=0.4.

By ‘curvature’, we mean the Gaussian curvature *K*=*κ*_1_*κ*_2_, the product of the largest and smallest normal curvatures of curves tangent to *S* at a specific point. The normal curvature is measured in the plane spanned by the tangent vector and the normal to the surface. The Gaussian curvature can be computed in terms of the first and second fundamental forms, and, for a surface of revolution, reduces to

5.14 $$K=\frac{\zeta^{\prime }(\rho^{\prime }\zeta^{\prime \prime }-\rho^{\prime \prime }\zeta^{\prime })}{\rho {\left({\left(\rho^{\prime }\right)}^{2}+{\left(\zeta^{\prime }\right)}^{2}\right)}^{2}}.$$

For the bean-shaped surface, we find

5.15 $$K=\frac{\left(2a\cos\theta -b)(2a\cos^{3}\theta -b\right)}{{\left(\cos^{2}\theta +\sin^{2}\theta {\left(2a\cos\theta -b\right)}^{2}\right)}^{2}},$$

showing that—if 2*a*>*b*—then *K*=0 at the ‘rim’ $\theta =\theta_{r}=\cos^{-1}(b/2a)$ (where the surface reaches its maximum height *a*+*b*^2^/(4*a*)) and also at the smaller angle $\theta =\theta_{m}=\cos^{-1}(\sqrt[{3}]{b/2a})$. For *θ*_*m*_<*θ*<*θ*_*r*_, the Gaussian curvature is negative.

**Remark**

For *a*≠0, it does not appear possible to obtain closed-form expressions for the various functions λ, *μ*, etc. Nevertheless, they can be obtained numerically to high precision. Special care is needed, however, to find the conformal factor λ(*θ*). From (4.17), we have

5.16 $$\frac{\lambda^{\prime }}{\lambda }=\frac{\rho^{\prime }-\sqrt{{\left(\rho^{\prime }\right)}^{2}+{\left(\zeta^{\prime }\right)}^{2}}}{\rho }.$$

The r.h.s. vanishes as *θ*→0 but is singular for *θ*→*π*. To obtain an accurate expression for λ, it is necessary to add and subtract (cos⁡θ−1)/sin⁡θ from the r.h.s., as this term can be integrated exactly and compensates the singularity at *θ*→*π*:

5.17 $$\log\lambda =\log(1+\cos\theta )+\int_{0}^{\theta }\left(\frac{\rho_{o}^{\prime }-\sqrt{{\left(\rho_{o}^{\prime }\right)}^{2}+{\left(\zeta_{o}^{\prime }\right)}^{2}}}{\rho_{o}}-\frac{\cos\theta_{o}-1}{\sin\theta_{o}}\right)d\theta_{o}.$$

Note that λ(0)=2 while λ(*π*)=0. The integral above and that to find *μ*(*θ*) from

5.18 $$\mu =-\int_{0}^{\theta }\rho_{o}\sqrt{{\left(\rho_{o}^{\prime }\right)}^{2}+{\left(\zeta_{o}^{\prime }\right)}^{2}}\;d\theta_{o}+C$$

(where we are free to choose *C* so that *μ*(*π*)=−*μ*(0)) are obtained using two-point Gaussian quadrature with 1800 equal divisions of *θ* between 0 and *π*, giving results accurate to one part in 10^14^ for moderate values of *a* and *b*. Once these functions are computed, all other functions can be found directly, without integration.

**Figure 3.**
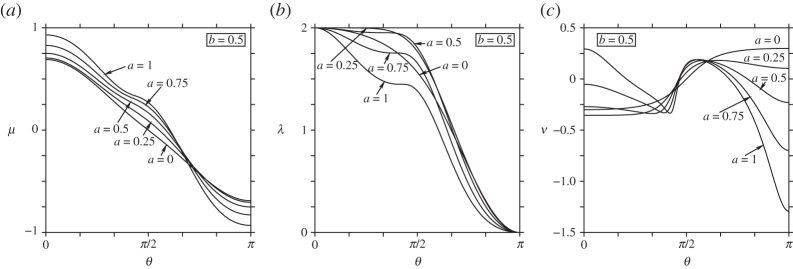
The form of various surface functions for the bean (here for fixed *b*=0.5): (*a*) the meridional coordinate *μ*(*θ*), (*b*) the conformal factor λ(*θ*) and (*c*) the rotation rate *ν*(*θ*) of a single vortex with *Γ*=2*π*.

We next illustrate the dependence of the functions λ(*θ*), *μ*(*θ*) and *ν*(*θ*) in (5.12) on the parameters *a* and *b*. Recall *ν*(*θ*) is the rotation rate of a single point vortex of strength *Γ*=2*π* about the axis of symmetry *z*. Figure 3 shows *μ*(*θ*), λ(*θ*) and *ν*(*θ*) for various values of *a* and for *b*=0.5. For *a*>0, the meridional coordinate *μ*(*θ*) is no longer symmetric about the ‘equator’ *θ*=*π*/2. An additional inflection occurs for *a*>0.25; this inflection arises from a region of negative Gaussian curvature *K*<0 located in a belt around the north pole; in general, this occurs when 2*a*>*b* (see discussion above). This inflection is also seen in the conformal factor λ(*θ*), which develops a short plateau before steeply dropping off for *θ*>*π*/2. The rotation rate *ν*(*θ*) like *μ*(*θ*) loses symmetry about the equator when *a*>0. Two zones of strong anti-cyclonic rotation develop for the larger values of *a*. The first zone is centred on the rim at $\theta =\cos^{-1}(b/2a)$, where the Gaussian curvature *K* changes sign. The second zone is centred on the south pole and is evidently associated with the increasing curvature there as *a* increases—note the similarity to the *b*=2 case in figure 2. Essentially, the region near the south pole is becoming locally prolate, inducing anti-cyclonic rotation about the axis of symmetry (or cyclonic rotation about the south pole, considering this pole as ‘up’) (figure 1*c*).

## 6. Stability of a ring of vortices

**Figure 4.**
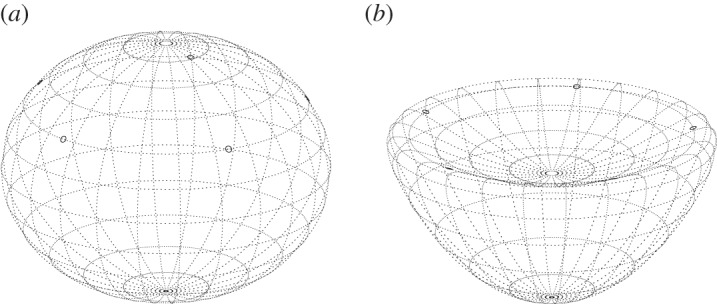
Polygonal relative equilibria of five identical vortices. (*a*) Ellipsoid of rotation with *a*=0 and *b*=0.8 and (*b*) bean-shape surface with *a*=0.6 and *b*=0.4.

To explore the effects of variable curvature on the dynamics of point vortices, we examine next the stability of a ring of vortices located at ‘co-latitude’ *θ* on a general surface of revolution (figure 4). We first consider linear stability, then illustrate several instabilities in the fully nonlinear dynamics. Specific results are presented for the ellipsoid and compared with the known results for a sphere [14].

### (a) Linear stability

A system of *n* identical vortices (*Γ*_*k*_=*Γ*_0_, *Γ*=*nΓ*_0_) initially lying on the co-latitude ring *θ*_*k*_=*θ* at equally spaced longitudes *ϕ*_*k*_=2*πk*/*n*, *k*=1,2,…,*n* rotates steadily about the north pole at a fixed angular velocity *ν* (it is said to be a ‘relative equilibrium’). The vortex motion is simply given by *ϕ*_*k*_(*t*)=2*πk*/*n*+*νt*, *θ*_*k*_(*t*)=*θ*. The angular velocity $\nu ={\dot{\phi }}_{k}$ can be calculated from $u_{k}/\sin\theta_{k}$ (for any *k*) using (4.19). Taking *Γ*_0_=2*π* without loss of generality, we obtain

6.1 $$\nu =\frac{n\beta }{\sin\theta }+\frac{\tau }{4\rho }\sum_{j=1}^{n-1}\frac{\lambda \xi^{\prime }+\lambda^{\prime }\xi -2\rho \rho^{\prime }\cos(2\pi j/n)}{\lambda \xi -\rho^{2}\cos(2\pi j/n)}.$$

Above, all surface functions (*β*,*τ*,…) are evaluated at *θ*. However, *ξ*=*ρ*^2^/λ so *λξ*=*ρ*^2^. Also, *λξ*′+λ′*ξ*=(*λξ*)′=2*ρρ*′, so the sum reduces simply to 2(*n*−1)*ρ*′/*ρ*. Therefore,

6.2 $$\nu =\frac{n\beta }{\sin\theta }+\frac{(n-1)\tau \rho^{\prime }}{2\rho^{2}}.$$

Using $\tau =1/\sqrt{{\left(\rho^{\prime }\right)}^{2}+{\left(\zeta^{\prime }\right)}^{2}}$ and the definition of *β* in (4.21), we obtain the final expression

6.3 $$\nu =\frac{1}{2\rho^{2}}\left[\frac{n\mu }{\mu (0)}-\frac{\rho^{\prime }}{\sqrt{{\left(\rho^{\prime }\right)}^{2}+{\left(\zeta^{\prime }\right)}^{2}}}\right].$$

When *n*=1, this reduces to the single-vortex rotation rate in (5.12). For a spherical surface, $\nu =(n-1)\cos\theta /2\sin^{2}\theta$, in agreement with that found in [14,18].

Linear stability is determined by displacing the vortices infinitesimally, taking *ϕ*_*k*_=2*πk*/*n*+*δϕ*_*k*_ and *θ*_*k*_=*θ*+*δθ*_*k*_, and solving the resultant linearized equations for *δϕ*_*k*_(*t*) and *δθ*_*k*_(*t*). Seeking eigen-solutions of the form $\delta \phi_{k}(t)=ℜ\{{\hat{\phi }}_{k}e^{\sigma t}\}$ and $\delta \theta_{k}(t)=ℜ\{{\hat{\theta }}_{k}e^{\sigma t}\}$, the objective is to find the growth rate *σ* (or frequency if *σ* is purely imaginary) by solving the resultant linear algebraic equations. As in [14], this procedure can be simplified considerably by exploiting the rotational symmetry of the basic state. This symmetry is apparent in the general form of the matrix eigen-problem,

6.4 $$\sigma \text{v}=\left(\begin{array}{cc} \text{A} & \text{B}\\ \text{C} & \text{D} \end{array}\right)\text{v},$$

where $\text{v}={\left({\hat{\phi }}_{1},\ldots ,{\hat{\phi }}_{n},{\hat{\theta }}_{1},\ldots ,{\hat{\theta }}_{n}\right)}^{T}$ and A, B, C and D are cyclic *n*×*n* matrices of the form

6.5 $$\text{A}=\left(\begin{array}{cccccc} a_{1} & a_{2} & a_{3} & \cdots & a_{n-1} & a_{n}\\ a_{n} & a_{1} & a_{2} & \cdots & a_{n-2} & a_{n-1}\\ a_{n-1} & a_{n} & a_{1} & \cdots & a_{n-3} & a_{n-2}\\ \cdots & \cdots & \cdots & \cdots & \cdots & \cdots \\ a_{2} & a_{3} & a_{4} & \cdots & a_{n} & a_{1} \end{array}\right).$$

As a result, the eigenvectors ***v*** correspond to special vortex displacements of the form

6.6 $${\hat{\phi }}_{k}=\hat{\phi }\;e^{2\pi ikm/n}\;\mathrm{and}\;{\hat{\theta }}_{k}=\hat{\theta }\;e^{2\pi ikm/n}\;(m=1,2,\ldots n).$$

Note, there are *m* distinct modes of displacement, and any general displacement can be represented as a linear combination of these modes. Substituting these forms for ${\hat{\phi }}_{k}$ and ${\hat{\theta }}_{k}$ into the matrix eigen-problem, we obtain a 2×2 problem for each mode *m*:

6.7 $$\begin{array}{rl} {\tilde{a}}_{m}\hat{\phi }+{\tilde{b}}_{m}\hat{\theta } & =\sigma \hat{\phi }\\ \text{and}\;{\tilde{c}}_{m}\hat{\phi }+{\tilde{d}}_{m}\hat{\theta } & =\sigma \hat{\theta }, \end{array}\}$$

where

6.8 $${\tilde{a}}_{m}=\sum_{j=1}^{n}a_{j}\;e^{2\pi i(j-1)m/n},$$

etc.

The calculation of the *a*_*j*_, *b*_*j*_, *c*_*j*_ and *d*_*j*_ is straightforward but laborious, so details are omitted. It turns out that all *a*_*j*_=*d*_*j*_=0, while, for the other coefficients, we obtain

6.9 $$b_{1}=\nu^{\prime }+\frac{n^{2}-1}{12\rho^{3}\tau };\;b_{j}=-\frac{1}{2\rho^{3}\tau (1-\cos(2\pi (j-1)/n))},\;j>1$$

and

6.10 $$c_{1}=\frac{(n^{2}-1)\tau }{12\rho };\;c_{j}=-\frac{\tau }{2\rho (1-\cos(2\pi (j-1)/n))},\;j>1,$$

where in *b*_1_

6.11 $$\nu^{\prime }=\frac{K-4n\pi /A}{2\rho \tau }-\frac{2\rho^{\prime }\nu }{\rho }$$

and *K* is the Gaussian curvature defined in (5.14). Then, using

6.12 $$\varpi_{mn}\equiv \sum_{j=1}^{n-1}\frac{\cos(2\pi mj/n)}{1-\cos(2\pi mj/n)}=\frac{n^{2}-1}{6}-m(n-m),$$

the coefficients ${\tilde{b}}_{m}$ and ${\tilde{c}}_{m}$ for each mode *m* simplify to

6.13 $${\tilde{b}}_{m}=b_{1}-\frac{\varpi_{mn}}{2\rho^{3}\tau }=\nu^{\prime }+\frac{m(n-m)\tau }{2\rho^{3}\tau };\;{\tilde{c}}_{m}=\frac{m(n-m)\tau }{2\rho }.$$

Putting this altogether, the growth rate of each mode *m* is just $\sigma =\sigma_{m}=\pm \sqrt{{\tilde{b}}_{m}{\tilde{c}}_{m}}$, or, using (6.11) and (6.13),

6.14 $$\sigma_{m}=\pm \frac{\sqrt{m(n-m)[m(n-m)+\rho^{2}(K-4n\pi /A-4\nu \rho^{\prime }\tau )]}}{2\rho^{2}},$$

where (*m*=1,2,…,*n*). In this expression, we have taken all vortex circulations *Γ*_*k*_ to be 2*π*. If we instead take *Γ*_*k*_=*Γ*_0_, the above expression must be multiplied by *Γ*_0_/2*π*. Note that the rotation rate *ν*, defined in (6.3) and appearing in (6.14), is dimensionless. Like *σ*_*m*_ the dimensional rotation rate is given by *ν* times *Γ*_0_/2*π*.

For the sphere, this reduces to the form derived in [14], namely

6.15 $$\sigma_{m}=\pm \frac{\sqrt{m(n-m)[m(n-m)-(n-1)(1+\cos^{2}\theta )]}}{2\sin^{2}\theta }.$$

Instability occurs if $m(n-m)>(n-1)(1+\cos^{2}\theta )$ or, equivalently, if

$$\cos^{2}\theta <\frac{m(n-m)}{n-1}-1.$$

This never occurs for *m*=1, *n*−1 or *n*. Hence, ring configurations of three or fewer vortices are stable on a sphere. For *n*=4, instability occurs for $\cos^{2}\theta <1/3$, i.e. in an equatorial belt extending between *θ*=54.7… to 125.2… degrees, and, as *n* increases, the instability region widens. For *n*≥7, instability occurs at all *θ*.

**Figure 5.**
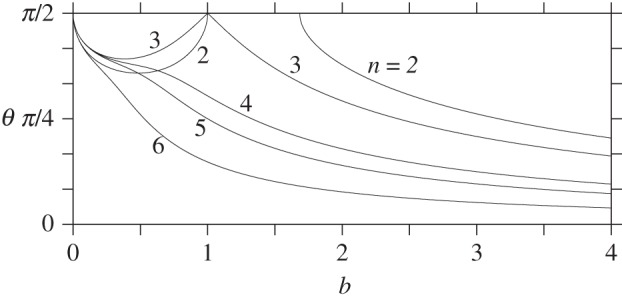
Instability domains for *n*=2–6 vortices, as a function of the aspect ratio of the ellipsoid *b*. For a given *n*, there is linear instability for co-latitudes between *θ*_*n*_(*b*) and *π*−*θ*_*n*_(*b*) (centred on the equator) wherever *θ*_*n*_(*b*)<*π*/2.

We now examine the ellipsoid. This surface is described by the functions ρ=sin⁡θ and ζ=bcos⁡θ (the sphere corresponds to the special case *b*=1). Figure 5 shows the domain of instability as a function of *b* for *n*=2–6 vortices (a single vortex is always stable while seven or more vortices are always unstable). Configurations with *θ*_*n*_(*b*)<*θ*<*π*−*θ*_*n*_(*b*) are unstable. Notably, as few as two vortices can be unstable, if sufficiently close to the equator, when *b*<1 (oblate surfaces) or when *b*>1.684541781 approximately (prolate surfaces). Three vortices always have a domain of instability near the equator except on the sphere *b*=1. Four to six vortices are unstable sufficiently near the equator for all *b*>0.

**Figure 6.**
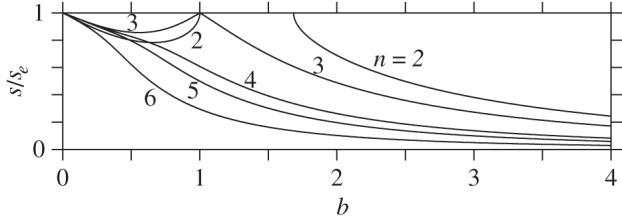
Same as figure 5 except that the arc-length ratio *s*(*θ*_*n*_(*b*))/*s*_*e*_ is shown. Here, *s*_*e*_ is the arc-length from the pole to the equator. The instability domain lies above the curves shown for each *n*, wherever *s*/*s*_*e*_<1. Note that *s*=0 corresponds to the north pole.

As *b*→0, the domain of instability appears to shrink to zero. However, d*θ* does not represent distance on an ellipsoid. An alternative is to use arc-length *s* measured from the north pole to the co-latitudes *θ*=*θ*_*n*_(*b*), normalized by the arc-length to the equator *s*_*e*_ (note: arc-length is equal to *E*(*θ* | 1−*b*^2^), where *E* is the incomplete elliptic integral of the second kind). Figure 6 shows the ratio *s*/*s*_*e*_ as a function of *b* for *n*=2–6 vortices. Comparing with figure 5, the domain of instability is seen to be larger for *b*>1 and smaller for *b*<1. The domain of instability is still seen to shrink to zero as *b*→0. Overall, this implies that vortices on oblate surfaces are generally more stable than vortices on prolate surfaces.

These stability results differ markedly from those reported in [22]. In that work, the effect of the Robin function—arising from the compensating uniform vorticity field associated with the Gauss condition (2.3)—was overlooked. The Robin function plays a key role in the dynamics when the sum of the vortex circulations is non-zero, as here.

### (b) Nonlinear evolution

**Figure 7.**
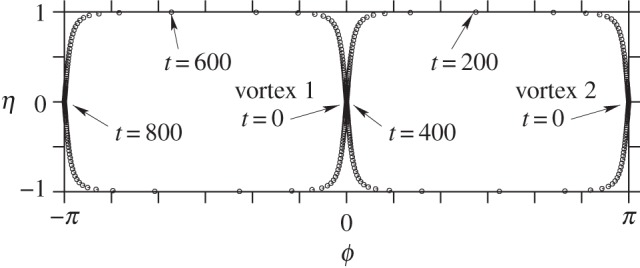
Vortex trajectories in the plane mapped by *ϕ* and *η*≡*μ*/*μ*(0) for two vortices initially starting at the equator of a slightly oblate ellipsoid, *b*=0.99. A circle is plotted each two units of time. See text for details.

We illustrate next several instabilities using the full nonlinear dynamics in (4.19). First, consider two equal vortices (*Γ*_1_=*Γ*_2_=2*π*) lying on the equator of a slightly oblate ellipsoid, *b*=0.99. According to the linear analysis, this state is unstable (figure 5). As an initial perturbation, we displace the vortex co-latitudes by ±0.001 (radians), keeping their longitudes at *ϕ*=0 and *π*. As shown in figure 7, the vortices move far from their equilibrium positions, passing close to the poles. In fact, vortex 1 remains in the northern hemisphere, while vortex 2 remains in the southern hemisphere. Initially, vortex 1 moves south then east, while vortex 2 moves north then west, in such a way that the two vortices remain on a diagonal passing through the equator at *ϕ*=*π*/2 (the trajectory resembles a great circle, though the surface is not a sphere). By *t*=400, the vortices have exchanged positions, then they repeat their polar excursions but with a displacement of *π* in *ϕ*. The whole evolution repeats periodically nearly every 800 time units. Notably, two vortices on a sphere are stable [14]. Only a slight oblateness therefore changes the stability properties markedly.

**Figure 8.**
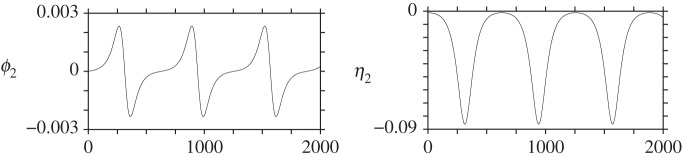
Evolution of *ϕ*_2_(*t*) (*a*) and *η*_2_(*t*) (*b*) for two vortices initially starting at the equator of a prolate ellipsoid with *b*=1.69.

Instability for two vortices also occurs on sufficiently prolate ellipsoids, *b*>1.6845…. Here, we illustrate a case with *b*=1.69, with the same initial conditions as before. This time the vortices do not move far from their initial positions, so it is more useful to plot *ϕ* and *η*=*μ*/*μ*(0) directly as a function of time. This is done in figure 8 for *ϕ*_2_(*t*) (left) and for *η*_2_(*t*) (right); vortex 1 exhibits a symmetric motion, with *ϕ*_1_=*π*−*ϕ*_2_ and *η*_1_=−*η*_2_. The strong, relative growth in *η*_2_ at early times is consistent with linear instability. However, the instability saturates at a small amplitude, then repeats itself periodically.

The periodic behaviour in the two cases illustrated is generic, given the number of degrees of freedom available. For two vortices, there are four coordinates. But for a general surface of revolution, there are two conserved quantities: the Hamiltonian *H* and the angular impulse *M*, (4.7). Moreover, *H* only depends on the longitudinal difference *α*=*ϕ*_2_−*ϕ*_1_. This leaves just one degree of freedom, and all that is possible is steady or periodic behaviour.

**Figure 9.**
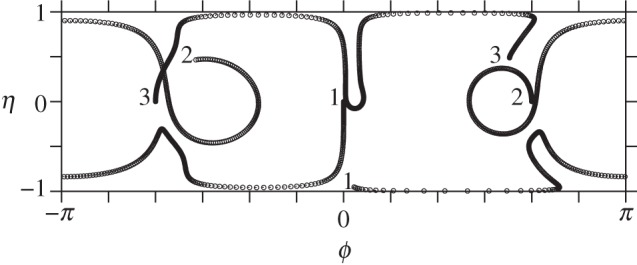
Vortex trajectories in the plane mapped by *ϕ* and *η*≡*μ*/*μ*(0) for three vortices initially starting at the equator of a slightly oblate ellipsoid, *b*=0.99. A circle is plotted each five units of time until *t*=2500. The initial and final locations of each vortex (1, 2 and 3) are marked. Note, *Γ*_1_=*Γ*_2_=*Γ*_3_=2*π*.

**Figure 10.**
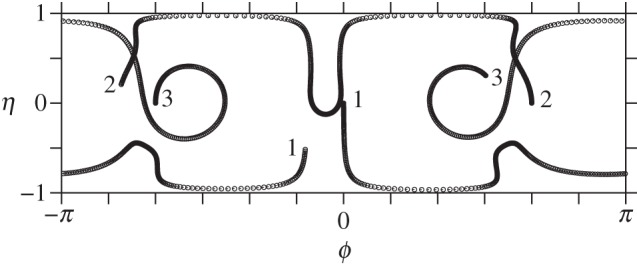
As in figure 9 but for a slightly prolate ellipsoid, *b*=1.01. The evolution does not become periodic at later times.

The motion of three vortices can be decidedly richer. Now there are three degrees of freedom, and therefore the potential for chaotic motion. On a sphere, though, three vortices are stable [14], even to finite-amplitude displacements [17]. Notably, the sphere has two additional conserved quantities (the other two components of the angular impulse), which constrain motions to be steady or periodic. However, a slight departure from a spherical surface leads to instability, both for *b*<1 and for *b*>1. This is illustrated in figures 9 and 10, respectively, for *b*=0.99 and for *b*=1.01—both slight deformations of a sphere. The vortex trajectories now exhibit a more irregular motion, potentially chaotic (this has not yet been investigated).

## 7. Conclusion

In this paper, we have developed general equations of motion for point vortices, singular concentrations of vorticity, moving on compact surfaces. We have sought to reveal the effects of surface geometry on vortex motion, in particular the self-induced motion associated with variable surface curvature. This self-induced motion stems from the mathematical requirement that the integral of the vorticity must vanish on a (differentiable) compact surface. As a result, a single point vortex must coexist with a background sea of uniform, non-zero vorticity (in fact, it could coexist with any background vorticity which satisfies this mathematical requirement, but a uniform background is the simplest as it reduces the dynamics to the motion of a single vortex). On surfaces of constant curvature like the sphere, the effect of this background sea is not directly apparent, but on surfaces of variable curvature, its effect is to induce vortex motion, at least when the sum of the vortex circulations is non-zero.

As an application, we investigated the linear and nonlinear stability of ‘vortex rings’, configurations of *n* equal vortices located in equilibrium on the same co-latitude and equally spaced in longitude, a problem first studied by Thomson [15] in the plane and later generalized to the sphere [14,19]. Here, we presented results for the ellipsoid of revolution, characterized by a vertical : horizontal aspect ratio *b*. We have found that vortex rings enjoy a wider range of stability on oblate surfaces (*b*<1) than on prolate ones (*b*>1). As few as two vortices may be unstable on oblate surfaces or sufficiently prolate ones, compared with four vortices for the sphere [14]. Our results correct the analysis in [22], in which the contribution by the Robin function was overlooked.

The formulation of vortex motion presented here appears to readily extend to vortex patches, uniform regions of vorticity bounded by material contours [12,31]. Vortex patch dynamics—contour dynamics [32]—requires only the Green function *G*, which we have derived for any differentiable compact surface of revolution. We hope to report on this extension in the near future.

## Footnotes

1 Without loss of generality, the coordinate system is chosen so that the undisturbed vortices lie on a latitude circle.

2 For example, on the unit sphere, we may take ***s***=(*ϕ*,*θ*), where *ϕ* and *θ* are the usual longitude and co-latitude coordinates. The corresponding particle position is then given by r=(cos⁡ϕ sin⁡θ,sin⁡ϕ sin⁡θ, cos⁡θ) in $R^{3}$.

3 Here we mean a scalar distribution, the normal component of the vorticity vector, whose tangential components are identically zero to ensure no normal flow component.

4 In the literature, the Robin function is often defined without the limiting process as a (symmetric) function of two variables, here ***s*** and ***s***_*j*_. This is sometimes referred to as the ‘Green function of the third kind’. Here, we only need the limiting form of *R* evaluated at a vortex position, and, for simplicity, we continue to call it the Robin function.

5 There is no continuous map from the whole closed surface *S* to the plane, since *S* is compact while the plane is not. By removing a point of *S* to create the punctured surface *S*_p_, we have actually made it topologically equivalent to the plane.

## Appendix A. Proof of proposition 3.2

Here, for a surface of revolution, we show how to evaluate $\bar{G}(\text{s})$ in (3.14), needed for *G*_*S*_ in (3.13). From (3.11) above,

A 1 $$\bar{G}(\text{s})=\frac{1}{A}\iint_{S_{p}}G_{S_{p}}(\text{s},{\text{s}}_{o})\;d\Omega_{{\text{s}}_{o}}=\frac{1}{A}\iint_{S_{p}}G_{P}(\Phi (\text{s}),\Phi ({\text{s}}_{o}))\;d\Omega_{{\text{s}}_{o}},$$

where the planar Green function *G*_*P*_(*Φ*(***s***),*Φ*(***s***_*o*_)) is given in (3.9). Since *ϕ* and *ϕ*_*o*_ only appear as the difference *α*=*ϕ*_*o*_−*ϕ* in *G*_*P*_, the function $\bar{G}$ in fact depends only on *θ*:

A 2 $$\bar{G}(\theta )=-\frac{1}{4\pi A}\int_{0}^{\pi }\int_{0}^{2\pi }\log[r^{2}(\theta )+r^{2}(\theta_{o})-2r(\theta )r(\theta_{o})\cos\alpha ]\mu^{\prime }(\theta_{o})\;d\theta_{o}\;d\alpha ,$$

where $\mu^{\prime }=-\rho \sqrt{{\left(\rho^{\prime }\right)}^{2}+{\left(\zeta^{\prime }\right)}^{2}}$. The coordinate *μ*(*θ*) is the natural meridional coordinate for the surface *S* since differential surface area can be expressed as d*Ω*_***s***_=−d*μ* d*ϕ*. Moreover, the area of the surface is given by

A 3 $$A=\iint_{S}d\Omega_{\text{s}}=2\pi (\mu (0)-\mu (\pi )),$$

a result which will prove useful just below. Returning to (A 2), the integral over *α* can be done immediately, leaving

A 4 $$A\bar{G}(\theta )=-\int_{0}^{\pi }\log r_{>}\mu^{\prime }(\theta_{o})\;d\theta_{o},$$

where $r_{>}=\max(r,r_{o})$. Splitting the integral into two parts, we have

$$A\bar{G}(\theta )=-\log r(\theta )\int_{0}^{\theta }\mu^{\prime }(\theta_{o})\;d\theta_{o}-\int_{\theta }^{\pi }\log r(\theta_{o})\mu^{\prime }(\theta_{o})\;d\theta_{o},$$

which, after integrating the second integral by parts, and the first exactly, we obtain

$$\begin{array}{rl} A\bar{G}(\theta ) & =-\log r(\theta ){\left[\mu (\theta_{o})-\mu (0)\right]}_{0}^{\theta }-{\left[\log r(\theta_{o})(\mu (\theta_{o})-\mu (\pi ))\right]}_{\theta }^{\pi }\\ & \;+\int_{\theta }^{\pi }(\mu (\theta_{o})-\mu (\pi ))\frac{r^{\prime }(\theta_{o})}{r(\theta_{o})}\;d\theta_{o}. \end{array}$$

Noting that *μ*(*θ*_*o*_)−*μ*(*π*)≈*μ*′′(*π*)(*θ*_*o*_−*π*)^2^/2 as *θ*_*o*_→*π*, there is no contribution from the upper limit *θ*_*o*_=*π* in the second term on the r.h.s., and the whole expression simplifies considerably:

A 5 $$\bar{G}(\theta )=\frac{1}{2\pi }\log[r(\theta )]+\frac{1}{A}\int_{\theta }^{\pi }(\mu (\theta_{o})-\mu (\pi ))\frac{r^{\prime }(\theta_{o})}{r(\theta_{o})}\;d\theta_{o},$$

where *μ*(0)−*μ*(*π*)=*A*/(2*π*) has been used. This function is potentially singular at *θ*=0 and *π*. At *θ*=0, it is easiest to work with the original expression for $\bar{G}$ in (A 4) evaluated at *θ*=0 exactly:

A 6 $$\bar{G}(0)=-\frac{1}{A}\int_{0}^{\pi }\log r(\theta_{o})\mu^{\prime }(\theta_{o})\;d\theta_{o}.$$

For a differentiable surface, $|\log r(\theta_{o})\mu^{\prime }(\theta_{o})|$ is finite for all *θ*_*o*_, hence $\bar{G}(0)$ is a constant: $\bar{G}$ is not singular as *θ*→0. However, as *θ*→*π*, the integral in (A 5) above goes to zero (it is straightforward to show that the integral is *O*((*θ*−*π*)^2^)), leaving

A 7 $$\bar{G}(\theta )\to \frac{1}{2\pi }\log[r(\theta )],$$

as *θ*→*π*. This is singular since r(θ)→∞ as *θ*→*π*. But this singularity is precisely what is needed to regularize the Green function *G*_*S*_ in (3.13) at *θ*=*π* (and at *θ*_*o*_=*π*, by symmetry).

## Appendix B. The Robin function

For a single vortex of circulation *Γ*_1_ located at a point ***s***=***s***_1_ on a compact surface *S*, the Hamiltonian (2.15) reduces to $-\Gamma_{1}^{2}R({\text{s}}_{1})/2$, where *R* is the Robin function defined in (2.13). The motion is then entirely controlled by the characteristics of *R*, characteristics which are directly acquired from the surface *S*. For a surface of revolution, we demonstrated at the end of §3 that *R* depends only on the co-latitude of the vortex, *θ*_1_. As a result, the vortex motion is constrained to move along a co-latitude circle, at the angular velocity *ν* given by (5.12).

On a sphere, we have shown that *R*=*constant*, so there is no self-induced vortex motion. The same is true for the non-compact hyperboloid studied in [20]. Both of these surfaces are special in that they have constant Gaussian curvature *K* (positive for the sphere and negative for the hyperboloid). The only other surface with constant curvature is the plane $R^{2}$, and again there is no self-induced motion.

However, compact surfaces are special in that they require the Gauss condition (2.3) for the solution of Poisson's equation (2.2). The simplest way of enforcing this condition is to add a compensating uniform vorticity distribution, as this does not add any degrees of freedom to the dynamics (material conservation of vorticity simply keeps the vorticity uniform). This background vorticity induces a flow, an axisymmetric one on a surface of revolution, which any point vortex must respond to. The only exception is when the sum of the vortex circulations is zero: then there is no background vorticity and no self-induced motion, regardless of the shape of *S*.

We note in passing that point vortex motion within a domain having exterior and/or interior boundaries exhibits analogous features. The Robin function *R*, defined as in (2.13), still induces the dynamics of a single vortex through the Hamiltonian $-\Gamma_{1}^{2}R({\text{s}}_{1})/2$. For example, for flow in the upper half-plane, it is simple to show that

B 1 $$R({\text{s}}_{1})=R(y_{1})=-\frac{1}{2\pi }\log(2y_{1}),$$

in terms of the usual planar coordinates (*x*_1_,*y*_1_). As a result, the flow is entirely in the *x* direction,

B 2 $$\frac{dx_{1}}{dt}=\frac{1}{\Gamma_{1}}\frac{\partial H}{\partial y_{1}}=-\frac{\Gamma_{1}}{2}\frac{\partial R}{\partial y_{1}}=\frac{\Gamma_{1}}{4\pi y_{1}}$$

as is well known.

## Appendix C. Invariance of Poisson's equation with a Dirac source

Consider a function $f\;:R^{2}\to R$. By definition, the Dirac distribution on $R^{2}$ satisfies

C 1 $$\iint_{R^{2}}\delta (\text{x}-{\text{x}}_{o})f(\text{x})\;d\Omega_{\text{x}}=f({\text{x}}_{o}),$$

where d*Ω*_***x***_ is the differential area element (or ‘area form’) of $R^{2}$, and ***x*** and ***x***_*o*_ are points in $R^{2}$. Now consider the conformal transformation of a punctured surface *S*_p_ to the plane $R^{2}$,

$$\begin{array}{rl} \Phi \;:\;S_{p} & ⟶R^{2}\\ \text{s}\; & ⟶\text{x}(\text{s}) \end{array}$$

with conformal factor λ^2^(***s***). This transforms (C 1) into

$$\iint_{S_{p}}\delta (\Phi (\text{s})-\Phi ({\text{s}}_{o}))f(\Phi (\text{s}))\lambda^{2}(\text{s})\;d\Omega =f(\Phi ({\text{s}}_{o})),$$

where d*Ω* is the area form of *S*, and ***s*** and ***s***_*o*_ are points on *S*. Defining *g*(***s***)=*f*(*Φ*(***s***)), this becomes

$$\iint_{S_{p}}\delta (\Phi (\text{s})-\Phi ({\text{s}}_{o}))g(\text{s})\lambda^{2}(\text{s})d\Omega =g({\text{s}}_{o}).$$

Hence, the Dirac distribution transforms according to

C 2 $$\delta (\Phi (\text{s})-\Phi ({\text{s}}_{o}))\lambda^{2}(\text{s})=\delta (\text{s}-{\text{s}}_{o}).$$

Now consider Poisson's equation with a Dirac source term on $R^{2}$:

$$\Delta_{\text{x}}G(\text{x},{\text{x}}_{o})=\delta (\text{x}-{\text{x}}_{o}).$$

By conformal transformation, this becomes (e.g. [33,34])

$$\frac{1}{\lambda^{2}(\text{s})}\Delta G(\Phi (\text{s}),\Phi ({\text{s}}_{o}))=\delta (\Phi (\text{s})-\Phi ({\text{s}}_{o})).$$

Using (C 2) to replace the right-hand side, we find

$$\Delta G(\Phi (\text{s}),\Phi ({\text{s}}_{o}))=\delta (\text{s}-{\text{s}}_{o}).$$

We can therefore conclude that Poisson's equation, with a Dirac distribution as its only source term, is conformally invariant. This is frequently taken for granted in other analyses of the problem (e.g. [35]).

Note that the above proof applies equally well for conformal transformations *Φ* from one planar 2D domain to another, e.g. as used to formulate the equations of motion in arbitrary 2D domains (see [8] and references therein). It is customary to use complex functions in such transformations, but the result is equivalent.

A reader less familiar with conformal transformations may wish to consider the one-dimensional case to gain a better intuition. In this case, for all suitable integrable functions f :R→R,

$$\int_{a}^{b}\delta (x-x_{o})f(x)\;dx=f(x_{o}),$$

if *x*_*o*_∈(*a*,*b*), by definition of the Dirac distribution *δ*. Now consider a coordinate transformation *y*(*x*) and its inverse *x*(*y*). The equation above then becomes

$$\int_{c}^{d}\delta (x(y)-x(y_{o}))f(x(y))x^{\prime }(y)\;dy=f(x(y_{o})),$$

where *c*=*y*(*a*) and *d*=*y*(*b*). Defining *g*(*y*)=*f*(*x*(*y*)), we obtain

$$\int_{c}^{d}\delta (x(y)-x(y_{o}))g(y)x^{\prime }(y)\;dy=g(y_{o}),$$

which implies that the Dirac distribution transforms according to

$$\delta (y-y_{o})=\delta (x(y)-x(y_{o}))x^{\prime }(y).$$

Hence, *x*′(*y*) plays the role of the conformal factor but in one dimension.
